# Intramolecular trimerization, a novel strategy for making multispecific antibodies with controlled orientation of the antigen binding domains

**DOI:** 10.1038/srep28643

**Published:** 2016-06-27

**Authors:** Ana Alvarez-Cienfuegos, Natalia Nuñez-Prado, Marta Compte, Angel M. Cuesta, Ana Blanco-Toribio, Seandean Lykke Harwood, Maider Villate, Nekane Merino, Jaume Bonet, Rocio Navarro, Clara Muñoz-Briones, Karen Marie Juul Sørensen, Kasper Mølgaard, Baldo Oliva, Laura Sanz, Francisco J. Blanco, Luis Alvarez-Vallina

**Affiliations:** 1 Department of Antibody Engineering, Leadartis SL, Ferraz 3, 28008 Madrid, Spain; 2 Molecular Immunology Unit, Hospital Universitario Puerta de Hierro Majadahonda, Manuel de Falla 1, 28222 Madrid, Spain; 3 Department of Engineering, Aarhus University, Gustav Wieds Vej 10, 8000 C Aarhus, Denmark; 4 Structural Biology Unit, CIC bioGUNE, Parque Tecnológico de Bizkaia, 48160 Derio, Spain; 5 Structural Bioinformatics Laboratory, Biomedical Informatics Research Unit, Parc de Recerca Biomèdica de Barcelona, 08003 Barcelona, Spain; 6 IKERBASQUE, Basque Foundation for Science, 48013 Bilbao, Spain

## Abstract

Here, we describe a new strategy that allows the rapid and efficient engineering of mono and multispecific trivalent antibodies. By fusing single-domain antibodies from camelid heavy-chain-only immunoglobulins (V_HHs_) to the N-terminus of a human collagen XVIII trimerization domain (TIE^XVIII^) we produced monospecific trimerbodies that were efficiently secreted as soluble functional proteins by mammalian cells. The purified V_HH_-TIE^XVIII^ trimerbodies were trimeric in solution and exhibited excellent antigen binding capacity. Furthermore, by connecting with two additional glycine-serine-based linkers three V_HH_-TIE^XVIII^ modules on a single polypeptide chain, we present an approach for the rational design of multispecific tandem trimerbodies with defined stoichiometry and controlled orientation. Using this technology we report here the construction and characterization of a tandem V_HH_-based trimerbody capable of simultaneously binding to three different antigens: carcinoembryonic antigen (CEA), epidermal growth factor receptor (EGFR) and green fluorescence protein (GFP). Multispecific tandem V_HH_-based trimerbodies were well expressed in mammalian cells, had good biophysical properties and were capable of simultaneously binding their targeted antigens. Importantly, these antibodies were very effective in inhibiting the proliferation of human epidermoid carcinoma A431 cells. Multispecific V_HH_-based trimerbodies are therefore ideal candidates for future applications in various therapeutic areas.

The development of hybridoma technology in 1975 by Kohler and Milstein^1^ provided an invaluable tool for the generation of highly specific monoclonal antibodies (mAb) with a plethora of applications in research, diagnosis, and therapy^2^. Up to date, forty-three mAbs have been approved by US or EU regulatory agencies for therapeutic use^3^. These molecules are generally well tolerated and constitute powerful treatment options for a variety of pathological conditions^4^. However, conventional bivalent monospecific mAbs have limitations, such as inadequate pharmacokinetics and tissue accessibility and unwanted Fc-mediated interactions, at least in some contexts^5^.

To overcome these handicaps, considerable efforts have focused on the development of next generation antibody-based therapeutics^6^. Conversion of monovalent antibody fragments [e.g. fragment antigen-binding (Fab), single-chain variable fragment (scFv), or single-domain antibody (sdAb)], into multivalent formats enhances functional affinity, decreases dissociation rates and improves biodistribution^5^. The most common strategies to produce multivalent formats have been the engineering of fusion proteins in which the antibody fragment makes a complex with oligomerization domains, and the generation of concatenated “tandem” subunit constructs^7^.

Recently, we have developed a technology platform for the rapid and efficient generation of multivalent antibodies, termed trimerbodies^8^,^9^,^10^. Engineered homotrimeric antibodies have been obtained by fusing scFv fragments with collagen-derived trimerization (TIE) domains, composed of the N-terminal trimerization region of collagen XVIII NC1 or collagen XV NC1 flanked by flexible peptide linkers^11^. Using this technology we have generated monospecific trivalent trimerbodies (N-trimerbodies or C-trimerbodies; 110 kDa) and monospecific or bispecific hexavalent trimerbodies (N/C-trimerbodies; 190 kDa). Trivalent and hexavalent scFv-based trimerbodies demonstrated excellent antigen binding capacity and multivalency *in vitro* and tumor-targeting efficacy *in vivo* in several mouse models of cancer^9^,^12^.

In the present study, we have generated N-terminal trimerbodies by fusing sdAbs from camelid heavy-chain-only immunoglobulins (V_HHs_) to the N-terminus of a TIE^XVIII^ domain. V_HH_ antibodies are of particular interest for protein engineering approaches. Despite their small-size (12–15 kDa) and strict monomeric behavior they possess affinities in the same range of conventional antibodies with paired V_H_/V_L_domains^13^,^14^. Furthermore, we present an approach to the rational design of multispecific tandem V_HH_-based trimerbodies with defined stoichiometry. Tandem trimerbodies were built by connecting with two additional glycine-serine-based linkers three V_HH_-TIE^XVIII^ modules on a single polypeptide chain. Recombinant V_HH_-based trimerbodies were efficiently secreted as soluble proteins by transfected human HEK-293 cells and were able to recognize their cognate antigen/s with high affinity and specificity. The strategy described herein can be used to efficiently produce multispecific molecules from pre-existent sdAbs. As this new antibody format can target two or more antigens it might have therapeutic potential in multiples diseases, simultaneously inhibiting different pathways involved in their etiopathogenesis as a mean**s** to avoid the appearance of resistance.

## Results

### Design and expression of V_HH_-based N-terminal trimerbodies

**Figure 1:**
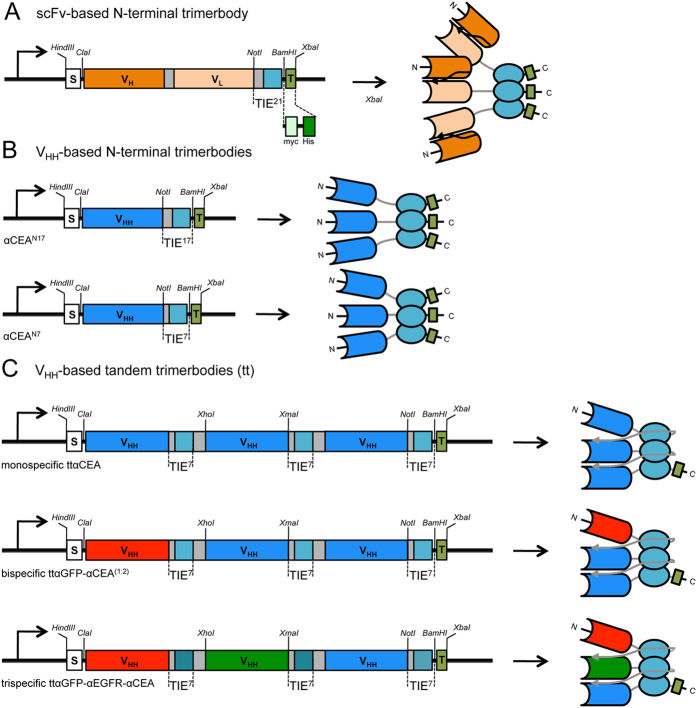
Schematic diagrams showing the genetic (left) and domain structure (right) of scFv-based (**A**) and V_HH_-based (**B,C**) trimerbodies. Conventional multi-chain trimerbodies (**A**,**B**) bear a signal peptide from the oncostatin M (S), a scFv gene (V_H_ and V_L_ domains joined by a flexible linker) (**A**) or a V_HH_ gene (**B**), and one TIE domain (teal boxes) flanked by flexible peptide linkers of different lengths (grey boxes), and epitope tags (T). The myc-tag (light green box) and the 6×His-tag (dark green box) were appended for immunodetection and affinity purification, respectively. Tandem V_HH_-based trimerbodies (**C**) bear a signal peptide from the oncostatin M (S), three V_HH_ genes and three TIE domains (teal boxes) flanked by 7-mer and 17-mer flexible peptide linkers (grey boxes), and epitope tags (T). Arrows indicate the direction of transcription.

**Figure 2:**
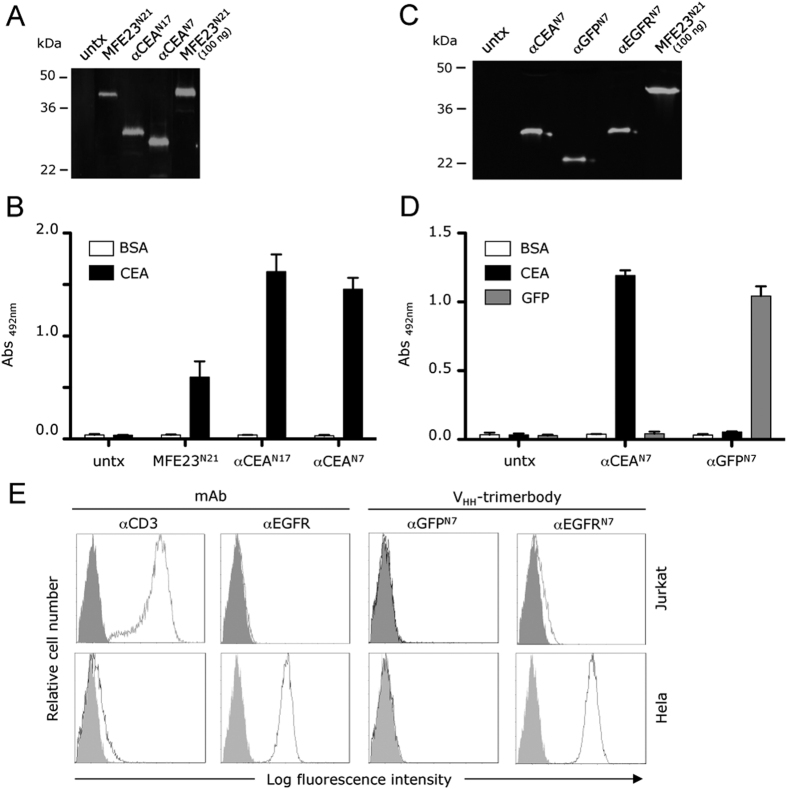
Characterization of recombinant trimerbodies. The presence of secreted scFv-based and V_HH_-based trimerbodies in the conditioned media from untransfected (untx) or transfected HEK-293 cells was demonstrated by western blot analysis, using 100 ng of purified anti-CEA scFv-based trimerbody (MFE23^N21^) as control (**A**–**C**). Migration distances of molecular mass markers are indicated (kDa). The blots were developed with anti-c-myc mAb, followed by incubation with an IRDye800-conjugated donkey anti-mouse IgG. The functionality of secreted trimerbodies was demonstrated, as described in the experimental procedures section, by ELISA against plastic immobilized CEA and GFP (**B**–**D**), and by FACS on EGFR-negative Jurkat cells and EGFR-positive HeLa cells (**E**), using 100 μl of neat supernatant from transiently transfected HEK-293 cells. Anti-CD3 (OKT3) and anti-EGFR (panitumumab) mAbs were used as controls on FACS studies.

In this study we generated N-terminal trimerbodies using sdAb fragments (V_HH_) as binding domains. We fused the CEA-specific CEA.1 V_HH_ to the N-terminus of a TIE^XVIII^ domain through flexible linkers of 17 or 7 residues (αCEA^N17^ or αCEA^N7^) (Fig. 1B). Both constructs were produced in HEK-293 cells more efficiently than the anti-CEA scFv-based trimerbody (MFE23^N21^) (Fig. 1A) (αCEA^N17^, 2.2 μg/ml × 10^5^ cells/48 hours; αCEA^N7^ 2.9 μg/ml × 10^5^ cells/48 hours; MFE23^N21^, 1.3 μg/ml × 10^5^ cells/48 hours). Western blot analysis under reducing conditions also showed a migration pattern of αCEA^N17^ and αCEA^N7^ consistent with the molecular weights calculated from their amino acid sequences (27.6 and 25.8 kDa, respectively) (Fig. 2A). ELISA analysis demonstrated that both αCEA^N17^ and αCEA^N7^ trimerbodies specifically recognize CEA (Fig. 2B).

To validate the ability of TIE domains to trimerize V_HH_ antibodies with different specificities, we designed N-terminal trimerbody constructs bearing an anti-GFP V_HH_ (αGFP) or an anti-EGFR V_HH_ (αEGFR) fused to the N-terminus of a TIE^XVIII^ domain through a 7-residue-long flexible linker (TIE^N7^). Both αGFP^N7^ and αEGFR^N7^ constructs were secreted by transfected human HEK-293 cells, to similar levels as the αCEA^N7^ (αGFP^N7^, 1.9 μg/ml × 10^5^ cells/48 hours; αEGFR^N7^ 2.1 μg/ml × 10^5^ cells/48 hours). Western blot analysis, under reducing conditions, demonstrated that αGFP^N7^ and αEGFR^N7^ trimerbodies were single-chain-type molecules with a migration pattern consistent with the molecular weights calculated from their amino acid sequences (22.4 and 27.1 kDa, respectively) (Fig. 2C).

ELISA analysis demonstrated that both secreted αCEA^N7^ and αGFP^N7^ trimerbodies specifically recognize their cognate antigens immobilized on plastic surface (Fig. 2D). The ability to detect antigen in a cellular context was studied by immunofluorescence labeling of human tumor cells (Jurkat or HeLa). Fluorescence staining was observed after incubation of the EGFR-expressing human cervix adenocarcinoma cell line HeLa with αEGFR^N7^, while no binding was detected for αGFP^N7^ (Fig. 2E). The EGFR-negative human T lymphoblastoid cell line Jurkat showed no binding of αEGFR^N7^ and αGFP^N7^ (Fig. 2E). These results demonstrated that the V_HH_-based trimerbodies recognized not only purified immobilized antigen, but also the antigen when expressed on the cell surface.

### Design and expression of tandem V_HH_-based trimerbodies

**Figure 3:**
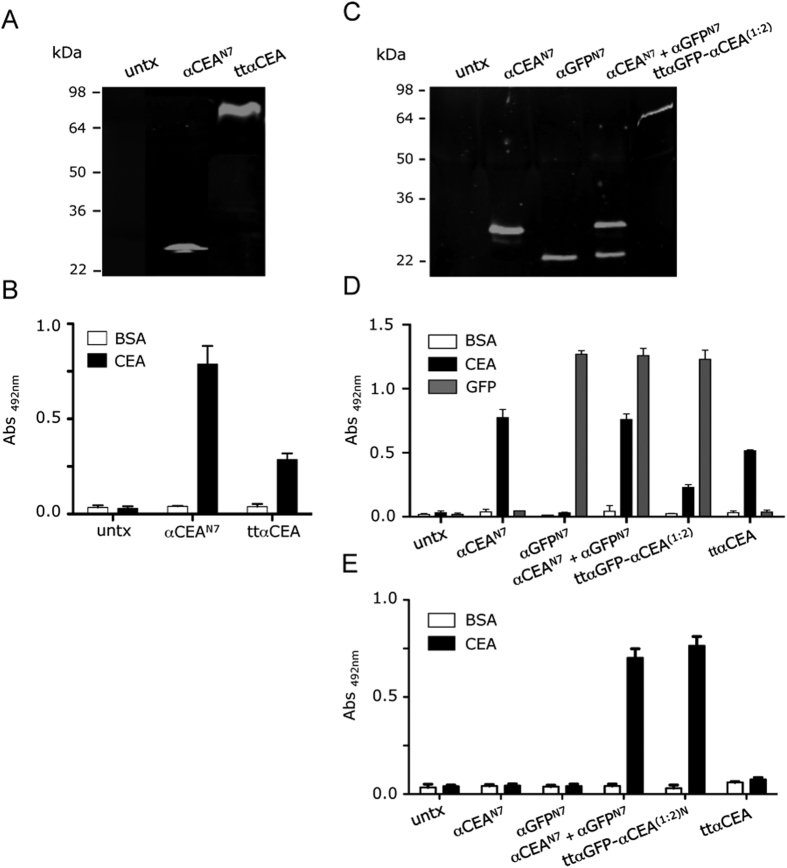
Characterization of recombinant tandem V_HH_-based trimerbodies. The presence of secreted multi-chain and tandem V_HH_-based trimerbodies in the conditioned media from gene-modified HEK-293 cells was demonstrated by western blot analysis (**A**–**C**). Migration distances of molecular mass markers are indicated (kDa). The blots were developed with anti-c-myc mAb, followed by incubation with an IRDye800-conjugated donkey anti-mouse IgG. The functionality of secreted trimerbodies was demonstrated, as described in the experimental procedures section, by ELISA against plastic immobilized CEA and GFP (**B**–**D**), using 100 μl of neat supernatant from transiently transfected HEK-293 cells. Simultaneous binding to the two targets was assessed by dual ELISA by direct immobilization of CEA, followed by 100 μl of neat supernatant from gene-modified HEK-293 cells and addition of GFP (**E**). Data shown are from a representative experiment out of three independent ones.

Next, we designed a V_HH_-based monospecific trimerbody in a single-chain format consisting of three αCEA^N7^ trimerbodies connected by two glycine-serine-based linkers on a single protein chain (V_HH_-based tandem trimerbodies; Fig. 1C). The anti-CEA tandem trimerbody (ttαCEA) was secreted as soluble functional protein by transfected HEK-293 cells (0.8 μg/ml × 10^5^ cells/48 hours). Western blot analysis demonstrated that under reducing conditions the migration pattern of the secreted ttαCEA is a single polypeptide chain with a molecular mass consistent with the 69.9 kDa calculated from its amino acid sequence (Fig. 3A). The functionality of ttαCEA was demonstrated by ELISA against plastic immobilized CEA (Fig. 3B). The differences in the ELISA signals may be a result of the number of c-myc tags, three copies in the conventional multi-chain trimerbody (αCEA^N7^) and one copy in the tandem single-chain trimerbody (ttαCEA) (Fig. 1B).

To further assess the multivalency and multispecificity of tandem V_HH_-based trimerbodies we designed a construct containing one copy of the αGFP^N7^ gene and two copies of the αCEA^N7^ gene connected by the described flexible linkers [ttαGFP-αCEA^(1:2)^] (Fig. 1C). This construct was produced as soluble functional protein by gene-modified HEK-293 cells (1.3 μg/ml × 10^5^ cells/48 hours). Western blot analysis under reducing conditions demonstrated that ttαGFP-αCEA^(1:2)^ is a single polypeptide chain with a molecular mass consistent with the 69.6 kDa calculated from its amino acid sequence (66.9 kDa without the signal sequence; Fig. 3C). The bispecificity of the ttαGFP-αCEA^(1:2)^ was analyzed by ELISA using immobilized human CEA and GFP. Whereas the ttαCEA showed specific binding to CEA, the ttαGFP-αCEA^(1:2)^ showed binding to both antigens (Fig. 3D). GFP has a molecular mass (27 kDa), which is significantly smaller than CEA (77 kDa). This determines a higher antigenic density in the GFP-coated ELISA plate, which allows to reach signal saturation even to the single c-myc-tagged ttαGFP-αCEA^(1:2)^. Furthermore, when conditioned medium from cotransfected (αGFP^N7^ and αCEA^N7^) or single-transfected (ttαGFP-αCEA^(1:2)^) HEK-293 cells was added to CEA-coated wells and, after washing, the CEA-bound trimerbodies were able to capture soluble GFP (Fig. 3E). Anti-CEA V_HH_-based trimerbodies (αCEA^N7^ and ttαCEA) were found to bind to CEA (Fig. 3D), but the bound trimerbodies did not capture soluble GFP (Fig. 3E).

**Figure 4:**
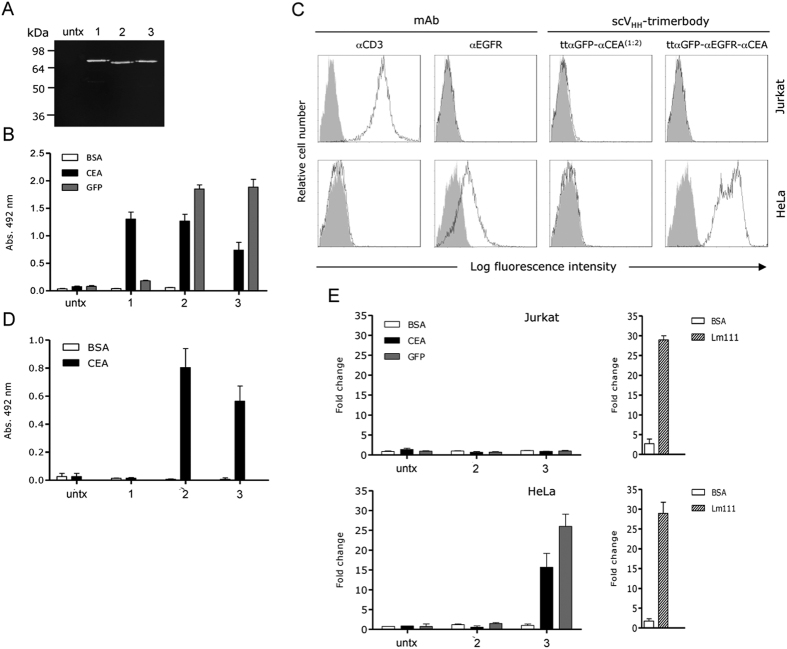
Characterization of recombinant tandem V_HH_-based trimerbodies: ttαCEA (1), ttαGFP-αCEA^(1:2)^ (**2**) and ttαGFP-αEGFR-αCEA (**3**). The presence of secreted tandem V_HH_-based trimerbodies in the conditioned media from gene-modified HEK-293 cells was demonstrated by western blot analysis (**A**). Migration distances of molecular mass markers are indicated (kDa). The blot was developed with anti-c-myc mAb, followed by incubation with an IRDye800-conjugated donkey anti-mouse IgG. The functionality of secreted tandem trimerbodies was demonstrated, as described in the experimental procedures section, by ELISA against plastic immobilized CEA and GFP (**B**), and by FACS on EGFR^-^ Jurkat cells and EGFR^+^ HeLa cells (**C**). Simultaneous binding to CEA and GFP was assessed by dual ELISA by direct immobilization of CEA, followed by 100 μl of neat supernatant from gene-modified HEK-293 cells and addition of GFP (**D**). Adhesion of EGFR^-^ Jurkat cells and EGFR^+^ HeLa cells to plastic-immobilized BSA, CEA or GFP, after incubation with 100 μl of neat conditioned media from gene-modified HEK-293 cells containing tandem V_HH_-based trimerbodies: ttαGFP-αCEA^(1:2)^ (**2**) and ttαGFP-αCEA-αEGFR (**3**). Adhesion of Jurkat and HeLa cells to plastic-immobilized laminin 111 (Lm111) was used as a control. Data are plotted as the log of fold change in adhesion relative to BSA. Data shown are from a representative experiment out of three independent ones.

In a further approach, we designed a tandem V_HH_-based trimerbody construct containing three different V_HH_ (αGFP^N7^, αEGFR^N7^ and αCEA^N7^) connected by the described flexible linkers [ttαGFP-αEGFR-αCEA] (Fig. 1C). The trispecific tandem trimerbody was secreted by transfected HEK-293 cells (1.0 μg/ml × 10^5^ cells/48 hours). Western blot analysis under reducing conditions revealed that ttαGFP-αEGFR-αCEA is a single polypeptide chain with a molecular mass consistent with the 72.2 kDa calculated from its amino acid sequence (68.5 without the signal sequence; Fig. 4A). Conditioned media from HEK-293 cells transfected with ttαGFP-αEGFR-αCEA recognized GFP and CEA immobilized on plastic (Fig. 4B). Tandem trimerbodies were further analyzed by flow cytometry for binding to EGFR-negative and EGFR-positive tumor cell lines. Fluorescence staining was observed after incubation of EGFR-expressing HeLa cells with ttαGFP-αEGFR-αCEA trimerbody, demonstrating its ability to detect the antigen in a cellular context (Fig. 4C). In contrast, incubation of HeLa cells with ttαGFP-αCEA^(1:2)^ trimerbody or incubation of Jurkat cells with ttαGFP-αEGFR-αCEA trimerbody revealed no staining (Fig. 4C). Conditioned medium from HEK-293 cells transfected with plasmids ttαGFP-αCEA^(1:2)^ or ttαGFP-αEGFR-αCEA was added to CEA-coated wells and, after washing, the CEA-bound trimerbodies were able to capture soluble GFP (Fig. 4D). V_HH_-based trimerbodies in the supernatant from HEK-293 transfected with plasmid encoding ttαCEA did not capture soluble GFP (Fig. 4D). To further assess the multivalency and multispecificity of tandem V_HH_-based trimerbodies we performed adhesion assays. As shown in Fig. 4E, EGFR-positive HeLa cells adhered to GFP- and CEA-coated wells after incubation with conditioned medium containing ttαGFP-αEGFR-αCEA. Moreover, trispecific tandem trimerbody was as efficient as laminin in supporting the adhesion of EGFR-positive cells. The adhesion of HeLa cells was specific since no adhesion of EGFR-negative Jurkat cells to GFP- and CEA-coated wells was detected (Fig. 4E). Furthermore, GFP- and CEA-coated wells, preincubated with conditioned medium from HEK-293 cells transfected with ttαGFP-αCEA^(1:2)^ plasmids did not support any significant cell adhesion (Fig. 4E).

### Purification and structural characterization of V_HH_-based trimerbodies

**Figure 5:**
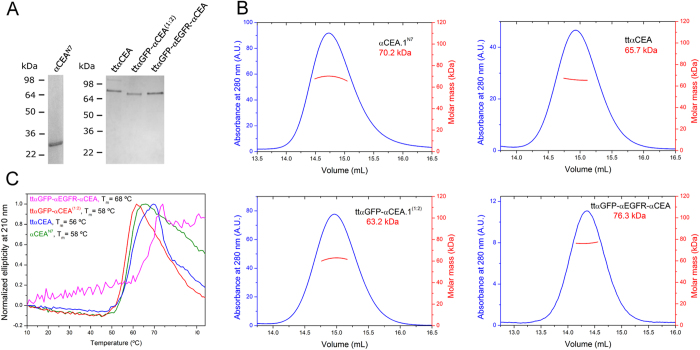
Structural characterization of purified multi-chain and tandem V_HH_-based trimerbodies. Reducing SDS-PAGE of the monospecific αCEA^N7^ and ttαCEA, the bispecific ttαGFP-αCEA^(1:2)^ and the trispecific ttαGFP-αCEA-αEGFR trimerbodies (**A**). Oligomeric analysis of the monospecific αCEA^N7^ and ttαCEA and the multispecific ttαGFP-CEA^(1:2)^ and ttαGFP-αEGFR-αCEA trimerbodies by SEC-MALLS with the indicated molecular masses measured at the center of the chromatography peaks (**B**). Tertiary structure analysis by thermal denaturation measured by the change in ellipticity at 210 nm for the four molecules (**C**). To facilitate comparison between curves of samples of different concentration and with different slopes in the baselines, the data were normalized from 0 (initial ellipticity) to 1 (the largest deviation from the initial value).

The αCEA^N7^, ttαCEA, ttαGFP-αCEA^(1:2)^ and ttαGFP-αEGFR-αCEA trimerbodies were purified from conditioned medium by immobilized metal ion affinity chromatography, which yielded proteins that were >90% pure, as estimated by visual inspection of reducing SDS-PAGE (Fig. 5A). The trimeric nature of the molecules was confirmed by SEC-MALLS measurements. The sample of αCEA^N7^ eluted from the size exclusion column as a major symmetric peak, and the mass calculated from the dispersed light at the center of the peak was 70.2 kDa (Fig. 5B). This value is smaller than the 77.3 kDa calculated for a trimer based on the amino acid sequence of this protein, but very close to the 69.2 kDa of a trimer calculated from the sequence excluding the N-terminal oncostatin M signal sequence. Mass spectrometry by matrix assisted laser desorption ionization confirmed the absence of the N-terminal 25 residues, suggesting that the signal sequence was cleaved during protein secretion. Purified tandem trimerbodies eluted from the size exclusion column as major symmetric peaks, with a very small portion of high molecular weight aggregates eluting at the exclusion volume of the column (Supplementary Fig. 1). The masses calculated from the dispersed light at the center of the peaks were 65.7, 63.2, and 76.3 kDa for ttαCEA, ttαGFP-αCEA^(1:2)^, and ttαGFP-αEGFR-αCEA respectively, close to the calculated values of 67.2, 66.9, and 68.5 kDa excluding the signal sequence (Fig. 5B).

The structure of the trimeric antibodies was investigated by circular dichroism. The spectra of the four proteins are very similar, with a minimum at around 218 nm (Supplementary Fig. 2), typical of proteins with predominantly β-sheet secondary structure. The trimerbodies are globally folded into stable three-dimensional structures, as seen by their cooperative thermal denaturations (Fig. 5C). The proteins show a major denaturation event with similar mid-point temperatures: 56 °C for ttαCEA and 58 °C both for αCEA^N7^ and ttαGFP-αCEA^(1:2)^. For ttαGFP-αCEA^(1:2)^, a second cooperative of smaller amplitude occurs with a mid-point temperature of 73 °C, possibly due to the unfolding of the anti-GFP moiety. For the trispecific trimerbody, a single broad denaturation event is observed centered at approximately 68 °C.

### Functional characterization of V_HH_-based trimerbodies

**Figure 6:**
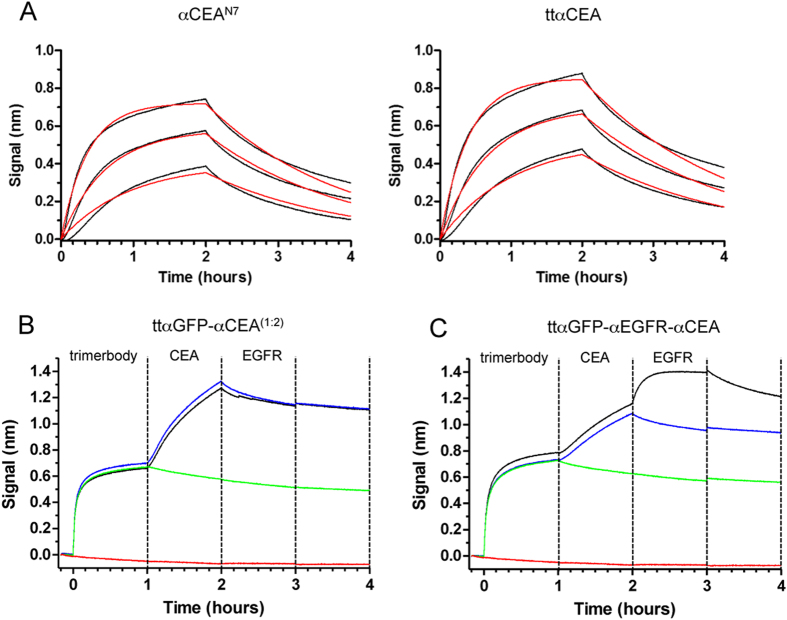
Functional characterization of purified multi-chain (αCEA^N7^) and tandem (ttαCEA, ttαGFP-αCEA^(1:2)^ and ttαGFP-αEGFR-αCEA) V_HH_-based trimerbodies. Biolayer interferometry (BLI)-derived sensorgrams from the interactions between immobilized αCEA^N7^ or ttαCEA trimerbody, and analyte CEA at 50, 25, or 12.5 nM. Experimental responses are traced in black, and fitting curves are in red (**A**). Concurrent binding of the ttαGFP-αCEA^(1:2)^ and ttαGFP-αCEA-αEGFR trimerbodies to antigens measured using BLI (**B**,**C**). In these experiments, GFP was immobilized on the biosensors. Simultaneous binding of the bispecific ttαGFP-αCEA^(1:2)^ trimerbody to GFP and human CEA (**B**). Concurrent binding of the trispecific ttαGFP-αCEA-αEGFR trimerbody to the three antigens: GFP, CEA and EGFR (**C**). Control biosensors were associated with trimerbody and CEA but not EGFR (blue traces), with trimerbody but neither antigen (green traces), or without trimerbody, with both CEA and EGFR (red traces).

The binding kinetics of the purified αCEA^N7^ and ttαCEA trimerbodies was studied using biolayer interferometry (BLI). As shown in Fig. 6A, the kinetic behavior of the αCEA^N7^/CEA and ttαCEA/CEA interactions were highly similar, indicating that the additional glycine-serine linkers introduced in the tandem trimerbody do not compromise its antigen binding capacity. Sensorgrams obtained using 50, 25, and 12.5 nM of CEA could be fit to a 1:1 model using very similar kinetic rate constants for both trimerbodies (Fig. 6A). The calculated dissociation constant (K_D_ = k_on_/k_off_) is 12 nM for both the αCEA^N7^/CEA and ttαCEA/CEA interactions (Supplementary Table S1). We next studied whether the binding sites of the purified ttαGFP-αCEA^(1:2)^ and ttαGFP-αEGFR-αCEA trimerbodies can bind concurrently to their cognate antigens. BLI-derived sensorgrams show a clear binding of both ttαGFP-αCEA^(1:2)^ and ttαGFP-αEGFR-αCEA trimerbodies to GFP-coated biosensors (Fig. 6B,C). Both trimerbody-loaded biosensors give additional binding curves upon addition of CEA. The ttαGFP-αEGFR-αCEA-loaded biosensors give a third binding curve upon addition of EGFR (Fig. 6C). In the absence of ttαGFP-αCEA^(1:2)^ and ttαGFP-αEGFR-αCEA trimerbodies, GFP-coated biosensors do not respond to CEA or hEGFR-Fc (Fig. 6B,C). These experiments show that the bispecific and trispecific V_HH_-based tandem trimerbodies can simultaneously bind to all their cognate antigens.

### Effect of purified tandem V_HH_-based trimerbodies on cell proliferation

**Figure 7:**
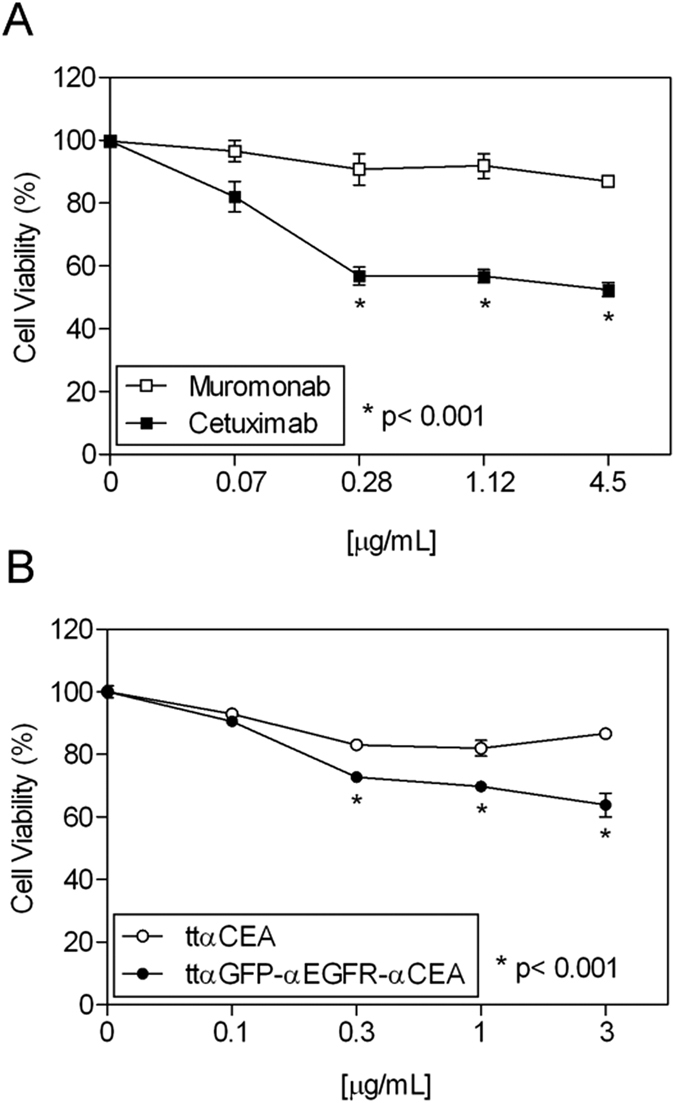
Biological activity of purified tandem V_HH_-based trimerbodies. A431 cells were treated with the indicated doses of cetuximab or muromonab (**A**), ttαCEA or ttαGFP-αCEA-αEGFR (**B**). Viable cells were measured in triplicates after 72 hours of treatment and plotted (mean ± SD) relative to untreated controls (*p < 0.001).

To assess the functionality of ttαGFP-αEGFR-αCEA in a biologically relevant context, *EGFR*-amplified A431 cells were treated with the purified protein. The anti-human EGFR mAb cetuximab was used as a positive control and the anti-human CD3ε mAb muromonab was used as a negative control. Cetuximab is a ligand-competitive inhibitor^15^; on the other hand, the parental anti-EGFR V_HH_EGa1 do not directly occlude the ligand-binding site on EGFR, but it prevents the EGFR extracellular region from adopting the extend conformation required for dimerization^15^. As shown in Fig. 7A, cetuximab, but not muromonab, inhibits A431 proliferation in a dose dependent manner. Similarly, treatment with ttαGFP-αEGFR-αCEA in the range 0.3–3 μg/ml resulted in a statistically significant decrease in proliferation (p < 0.001), indicating that in the tandem trimerbody the internal anti-EGFR V_HH_domain is highly efficient in blocking EGFR activation (Fig. 7B). As expected, the purified ttαCEA trimerbody did not inhibit the proliferation of A431cells.

### Serum stability of V_HH_-based trimerbodies

The serum stability was studied by incubating the purified multi-chain and tandem trimerbodies in human serum at 37 °C, for prolonged periods of time. The binding activity of the sample at 0 hours was set as 100% in order to calculate the time corresponding to percentage decay in binding activity. The multi-chain αCEA^N7^ and the tandem ttαCEA and ttαGFP-αCEA^(1:2)^ trimerbodies had similar stability, retaining more than 95% of the initial CEA- and GFP-binding activity after 96 hours of incubation (Supplementary Fig. 3).

### Analysis of the active surface

**Figure 8:**
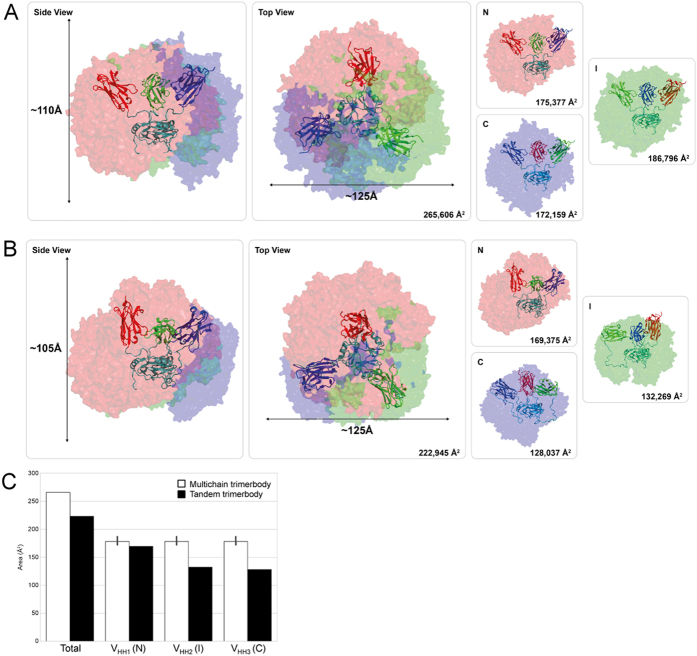
Representation of the putative surfaces accessible to the V_HH_ domains of the multi-chain αCEA^N7^ (**A**) and the tandem ttαCEA trimerbodies (**B**). For all structures represented, trimerization domains and linkers are depicted in teal, N-terminal V_HH_ (N) in red, internal V_HH_ (I) in green and C-terminal V_HH_ (C) in blue. For αCEA^N7^ N, I and C arbitrarily design one of the three identical chains just for comparison purposes. The surface area represented in each image is stated in its corresponding bottom-right corner. Side (left) and top (middle) representation of the overlapped surface explored by the three V_HH_ domains. A representation of the area explored for each V_HH_ domain can be seen together with its total surface area in the right. The final surface is calculated as the sum of all the volumes into one, thus the area is far smaller that the sum of the individual areas. (**C**) Representation of the difference in explored surface between the multi-chain (white bars) and the tandem trimerbody (black bars). N, I and C are exactly the same when related to the multi-chain trimerbody, thus, their explored surface is represented as the average with the standard error.

A comparative analysis of the positions that the V_HH_ domains can putatively explore around the trimerization domain was performed for both the multi-chain and the tandem trimerbody. Regardless of the presence of the additional linkers, the sterically allowed spatial distribution of the V_HH_ domains have an oblate shape, but slightly bigger for the multi-chain trimerbody with approximately 125 and 110 Å axes, than for the tandem trimerbody, with 125 and 105 Å axes (Fig. 8). The tandem V_HH_-based trimerbody is, on average, more compact, likely due to the restrictions in movement imposed by the extra linkers on the internal and C-terminal V_HH_ domains, but the difference is small (84% surface area as compared with the multi-chain trimerbody). However, the relative orientation of the V_HH_ domains is severely restricted in both trimerbodies, with defined regions on the surface predominantly occupied by each of the three V_HH_ domains (Fig. 8). As the multi-chain trimerbody contains three identical segments, each one of the V_HH_ domains explores similar surface areas. In the tandem trimerbody, however, the internal and C-terminal V_HH_ domains are linked to TIE trimerization domains by two linkers, but only one in the case of the N-terminal V_HH_ domain. As a consequence, the surface area sampled by the N-terminal V_HH_ domain is approximately 30% larger than that sampled by anyone of the other two (which sample approximately the same surface area).

## Discussion

In this study, we used the trimerbody technology platform for the production of trivalent V_HH_-based molecules. We fused V_HH_ antibodies with different specificities to the N-terminus of a human TIE^XVIII^ domain using linker sequences of different lengths. All the V_HH_-based trimerbodies were expressed in functional active form from conditioned medium of transfected HEK-293 cells, and importantly their production levels were higher than those of scFv-based trimerbodies with the same configuration. The purified V_HH_-based molecules were trimeric in solution and shown specific binding to their cognate antigens.

Furthermore, we demonstrated that multispecific molecules can be generated by co-transfecting HEK-293 cells with plasmids encoding several V_HH_-based trimerbodies with different specificities. However, assuming that the rate of synthesis is the same for all the proteins and that trimerization happens randomly one would expect 10% of the total trimerbody to be trispecific. Therefore, this strategy would require the isolation of the trimerbody with the desired V_HH_ combination, for instance by means of three purification steps based on three orthogonal affinity tags. Such a process may be feasible but not efficient.

Taking advantage of the small size and favorable biophysical properties of V_HH_ antibodies^13^,^14^ and human TIE^XVIII^ domain^16^, we present an approach to rationally design multispecific V_HH_-based N-trimerbodies with defined stoichiometry. We built tandem trimerbodies by connecting with additional glycine-serine-based linkers the C-terminus of the N-terminal and central TIE domain with the N-terminus of the central and C-terminal V_HH_ antibodies. Recombinant monospecific and multispecific (bi and trispecific) tandem V_HH_-based trimerbodies were efficiently secreted as soluble proteins by transfected human HEK-293 cells, and were easily purified using standard chromatographic methods. The purified tandem trimerbodies were highly homogeneous non-aggregating molecules in solution, as unambiguously shown by the light scattering measurements. The intramolecular assembly versus intermolecular assembly during the folding of the chains in the cell could be favored by the design of the linkers connecting the trimerization and the V_HH_ domains. Alternatively, intermolecular assemblies may be formed but are not secreted. The high level of protein production, however, suggests that intramolecular trimerization is favored.

The purified monospecific and multispecific tandem V_HH_-based trimerbodies were very efficient at recognizing antigen. Biolayer interferometry analysis revealed that the trispecific ttαGFP-αEGFR-αCEA tandem trimerbody was capable of binding concurrently to three antigens, indicating that each domain independently binds to its cognate antigen. Importantly, this molecule recognized surface EGFR as efficiently as the anti-EGFR mAb panitumumab, and it was very efficient and specific at inducing cell adhesion of EGFR-expressing cells when pre-incubated on plastic-bound CEA or plastic-bound GFP, but not on plastic-bound BSA. Furthermore, the purified ttαGFP-αEGFR-αCEA trimerbody was shown to block EGFR proliferation almost as efficiently as cetuximab in A431 cells. One could speculate that for some applications where a strong cross-linking of cell surface receptors is necessary, the presence of two V_HH_ domains with limited mobility could be favorable. The flexible linkers in the trimerbodies are long enough to allow the V_HH_ domains to access a large surface area around the trimerization domain but short enough to restrict their relative orientations, and in the tandem trimerbody, the additional linkers reduced the surface accessible to the central and C-terminal domains. Therefore, this design allows for tuning the relative mobility and orientation of the individual V_HH_ domains.

Monovalent V_HH_ antibodies are not optimal for the *in vivo* therapeutic applications. They have an *in vivo* half-life of approximately 1.5 h in blood^17^, and it has been demonstrated that *in vivo* non-equilibrium environments, even high-affinity monovalent interactions tend to have fast dissociation rates, providing modest retention times on the target antigen^18^. For therapeutic applications, it is therefore, desirable to engineer monovalent V_HH_ antibodies into multivalent molecules, with higher affinity, slower dissociation rates, and prolonged serum half**-lives^18^. Furthermore, multimerization allows the generation of multispecific molecules, which can enhance their tissue specificity and provide antibodies with novel functionality^7^. Although several methods have been devised for the generation of multivalent and multispecific single-domain antibodies, such as linear gene fusions^19^,^20^,^21^, self-associating peptides^22^,^23^ and protein domains^24^,^25^, the common strategy is based on the use of flexible peptide linkers. However, even bi- or trivalent tandem V_HH_ antibodies are below the threshold for first-pass renal clearance (about 60 kDa) and will therefore have a very short *in vivo* half-life. For this reason bivalent or trivalent tandem molecules containing an anti-human serum albumin V_HH_ antibody have been generated to extend the *in vivo* half-life of these molecules^26^.

Here we demonstrate for the first time that by inserting internal trimerization domains in a tandem V_HH_-based construct we can control the stoichiometry, the orientation, and the homogeneity of the species in solution. Tandem V_HH_-based trimerbodies were also characterized by a good thermostability, and a high resistance to serum proteases. These favorable properties and the intermediate molecular weight (65–70 kDa), makes the tandem V_HH_-based trimerbodies ideal candidates for *in vivo* cancer therapy. It is tempting to speculate that by modifying the length and amino acid composition of linkers connecting the trimerization domains we could fine-tune the geometry of tandem trimerbodies. Furthermore, by adding furin cleavage sites at both ends of the extra linker 1 and/or 2 we could restore the mobility of the internal and C-terminal V_HH_ domains, given that cleavage at both furin sites would lead to the removal of the additional peptide connectors^27^.

Multispecific tandem V_HH_-based trimerbodies may have applications for the dual targeting of two receptors for cancer therapy, the rationale design of more efficient multiparatopic molecules^28^, the development of immune cell recruitment strategies and the development of improved agonistic reagents for immunotherapy^29^. Other possible applications of V_HH_-based trimerbodies are the targeted delivery of a therapeutically active moiety, by fusing cytokines, toxins or enzymes to the C-terminus of the TIE^XVIII^ domain^10^, and the development of more effective trapping agents for the simultaneous neutralization of different angiogenic factors or disease-modulating cytokines.

## Materials and Methods

### Reagents and antibodies

Bovine serum albumin (BSA) (catalog no. A9418), pooled normal human serum (catalog no. H4522) and laminin-111 (Lm111) extracted from the Engelbreth-Holm-Swarm mouse tumor (catalog no. L2020) were from Sigma-Aldrich (St. Louis, MO, USA). Native human carcinoembryonic antigen (CEA) (catalog no. 219369) and recombinant green fluorescent protein (GFP) (catalog no. 14–392) expressed in *E. coli*, were from Merck Millipore (Billerica, MA, USA). The human EGFR-Fc chimera (catalog no. 10001-H02H-50) was from Sino Biological (Beijing, P.R.China). The mAbs used included: mouse anti-c-myc clone 9E10 (catalog no. ab32) (Abcam, Cambridge, UK), mouse anti-human CD3ε clone OKT3 muromonab (Ortho Biotech, Bridgewater, NJ, USA), chimeric mouse/human anti-human epidermal growth factor receptor (EGFR) cetuximab (Merck KGaA, Darmstadt, Germany) and fully human anti-human EGFR panitumumab (Amgen, Thousand Oaks, CA, USA). The polyclonal antibodies included: rabbit anti-GFP (catalog no. ab6556) (Abcam); phycoerytrin (PE)-conjugated goat F(ab′)_2_ fragment anti-mouse IgG, Fc specific, (catalog no. 115–116–146) (Jackson Immuno Research, Newmarket, UK); PE-conjugated goat F(ab′)_2_ fragment anti-human IgG (H&L) (catalog no. ab7006) (Abcam); horseradish peroxidase (HRP)-conjugated goat anti-mouse IgG, Fc specific, (catalog no. A0168) (Sigma-Aldrich); HRP-conjugated goat anti-rabbit IgG (catalog no. A8275) (Sigma-Aldrich); and IRDye800 conjugated donkey anti-mouse IgG (H&L) (catalog no. 610–732–002) (Rockland Immunochemicals, Gilbertsville, PA, USA).

### Cells and culture conditions

HEK-293 (catalog no. CRL-1573), HeLa (catalog no. CCL-2) and A431 (catalog no. CRL-1555) cells were cultured in Dulbecco’s modified Eagle’s medium (DMEM) (Lonza, Walkersville, MD, USA) supplemented with 2 mM L-glutamine, 10% (vol/vol) heat inactivated Fetal Calf Serum (FCS) and antibiotics (all from Life Technologies, Carlsbad, CA, USA) referred as to DMEM complete medium (DCM), unless otherwise stated. Jurkat clone E6–1 cells (TIB-152) were maintained in RPMI-1640 (Lonza) supplemented with 2 mM L-glutamine, heat-inactivated 10% FCS. All of these cell lines were obtained from the American Type Culture Collection (Rockville, MD, USA) and were routinely screened for the absence of mycoplasma contamination by PCR using the Mycoplasma Plus TM Primer Set (Stratagene, Cedar Creek, TX, USA).

### Construction of expression vectors

The mammalian expression vector pCEP4-MFE23-NC1^ES-^ encoding the CEA-specific MFE23 scFv-based N-terminal trimerbody has been previously described^9^. To construct the plasmids pCR3.1-CEA.1-TIE^17^ and pCR3.1-CEA.1-TIE^7^, two synthetic genes encoding the CEA-specific CEA.1 V_HH_ gene^30^ fused by a 17-mer or a 7-mer flexible linker to the N-terminus of the human TIE^XVIII^ domain (CEA.1-TIE^17^ and CEA.1-TIE^7^) were synthesized by Geneart AG (Regensburg, Germany) and subcloned as *Not*I/*Bam*HI into the vector pCR3.1-L36^31^. To generate the plasmid pCR3.1-αGFP-TIE^7^ the DNA fragment encoding the anti-GFP V_HH_ (αGFP)^32^ was PCR amplified from pNVgfp^33^, with primers *Cla*GFP (5′-TGTTGCGGCCGCTAGGGAGACGGTGACCTGG-3′) and *Not*GFP (5′-GCCACAT CGATGGCTCAGGTGCAGCTGGTG-3′). The *Cla*I/*Not*I-digested PCR fragment was ligated into the *Cla*I/*Not*I-digested backbone of plasmid pCR3.1-CEA.1-TIE^7^. To generate the plasmid pCR3.1-EGa1-TIE^7^ the DNA fragment encoding the anti-EGFR V_HH_ (EGa1)^34^ was PCR amplified from pNVEGa1, with primers *Cla*EGFR (5′-GCATGATCGATGATGGCTCAGGTGCAGCTCA-3′) and *Not*EGFR (5′-TTGTGCG GCCGCTGAGGAGACGGTGACCTGGGT-3′). The *Cla*I/*Not*I-digested PCR fragment was ligated into the *Cla*I/*Not*I-digested backbone of plasmid pCR3.1-CEA.1-TIE^7^. To generate the single-chain monospecific anti-CEA V_HH_-based trimerbody expression vector, a synthetic gene encoding two CEA.1-TIE^7^ constructs connected by a 17-mer flexible linker (^17^CEA.1^7^TIE^17^CEA.1^7^TIE) was synthesized by Geneart AG. The *Not*I cleaved fragment of this gene was ligated into pCR3.1-CEA.1-TIE^7^ to obtain the plasmid pCR3.1-scCEA.1-TIE. To generate the single-chain bispecific [αGFP x αCEA (x2)] V_HH_-based trimerbody expression vector, the *Not*I cleaved ^17^CEA.1^7^TIE^17^CEA.1^7^TIE gene was ligated into the *Not*I-digested backbone of plasmid pCR3.1-αGFP-TIE^7^ to obtain the plasmid pCR3.1-scαGFP-CEA.1(x2)-TIE. To generate the single-chain trispecific anti-GFP x anti-EGFR x anti-CEA V_HH_-based trimerbody expression vector the DNA fragment encoding the EGa1 V_HH_ was PCR amplified from pNVEGa1, with primers *Xma*EGFR (5′-GCATGCTCGAGGTATGG CTCAGGTGCAGCTCA-3′) and *Xho*EGFR (5′-TTCCCGGGTGAGGAGACGGTGAC CTGGGTCC-3′). The *Xho*I/*Xma*I-digested PCR fragment was ligated into the *Xho*I/*Xma*I-digested backbone of plasmid pCR3.1-scαGFP-CEA.1(x2)-TIE, to obtain the plasmid pCR3.1-scαGFP-EGa1-CEA.1-TIE. The sequences were verified using primers FwCMV (5′-CGCAAATGGGCGG TAGGCGTG-3′) and RvBGH (5′-TAGAA GGCACAGTCGAGG-3′).

### Expression and purification of recombinant antibodies

HEK-293 cells were transfected with the appropriate expression vectors using calcium phosphate^35^ and selected in DCM with 500 μg/ml G-418 (Sigma-Aldrich) to generate stable cell lines. Supernatants from transiently and stably transfected cell populations were analyzed by ELISA, western blotting and FACS. Stably transfected cell lines were used to collect serum free conditioned medium that was dialyzed against PBS (pH 7.4) and loaded onto a HisTrap HP 1 ml column using and ÄKTA Prime plus system (GE Healthcare, Uppsala, Sweden). The purified proteins were dialyzed against PBS and stored at −80 °C.

### Western blotting

Samples were separated under reducing conditions on 12% Tris-glycine gels and transferred to nitrocellulose membranes (Life Technologies) and probed with anti-c-myc mAb, followed by incubation with an IRDye800-conjugated donkey anti-mouse IgG. Visualization and quantitative analysis of protein bands were carried out with the Odyssey infrared imaging system (LI-COR Biosciences, Lincoln, NE, USA).

### ELISA

The ability of V_HH_-based trimerbodies to bind purified antigens (CEA or GFP) was studied by ELISA as described^9^. Briefly, Maxisorp (NUNC Brand Products, Roskilde, Denmark) plates were coated with CEA (0.3 μg/well) or GFP (0.5 μg/well) and after washing and blocking with 200 μl 5% BSA in PBS, 100 μl of purified protein solution, or 100 μl of neat supernatant from transiently or stably transfected HEK-293 cells, were added and incubated for 1 hour at room temperature. After three washes, 100 μl of anti-c-myc mab were added followed by 100 μl of HRP-conjugated goat anti-mouse IgG were added for 1 h at room temperature, after which the plate was washed and developed. To demonstrate the simultaneous reactivity of the two antigen specificities present in the bispecific and in the trispecific tandem trimerbody a dual ELISA was performed. Briefly, Maxisorp plates were coated with CEA (0.3 μg/well) and after washing and blocking, 100 μl of purified protein solution, or 100 μl of neat supernatant from transiently or stably transfected HEK-293 cells, were added and incubated for 1 hour at room temperature. After washing, 100 μl of recombinant GFP (10 μg/ml) were added followed by 100 μl of rabbit anti-GFP antibody. After washing, 100 μl of HRP-conjugated goat anti-rabbit IgG were added for 1 hour at room temperature, after which the plate was washed and developed.

### Flow cytometry

The ability of V_HH_-based trimerbodies to bind to cell surface EGFR was studied by FACS as described previously^10^. Briefly, HeLa or Jurkat cells were incubated with supernatants or purified trimerbodies (10 μg/ml) and anti-c-myc mAb for 30 min. After washing, the cells were treated with appropriate dilutions of PE-conjugated goat F(ab′)_2_ anti-mouse IgG. The samples were analyzed with a Beckman-Coulter FC-500 Analyzer (Coulter Electronics, Hialeah, FL, USA). Anti-CD3 (OKT3, mouse IgG_2a_) and anti-EGFR (panitumumab, human IgG_2_) mAbs were used as controls on FACS studies, using appropriate dilutions of PE-conjugated goat F(ab′)_2_ anti-mouse IgG and PE-conjugated goat F(ab′)_2_ anti-human IgG, respectively.

### Cell Adhesion Assay

96-well microtiter plates (Corning Costar, Cambridge, MA, USA) were coated overnight at 4 °C with CEA (2 μg/well), GFP (0.5 μg/well) or Lm111 (1 μg/well) and after washing and blocking with 200 μl 3% BSA-DMEM for 1 hour at 37 °C, 100 μl of supernatant from transfected HEK-293 cells were added for 1 hour at 4 °C. After washing aliquots of 5 × 10^4^ Jurkat or HeLa cells were loaded per well in serum-free medium and incubated for 30 minutes in humidified 5% CO_2_ atmosphere at 37 °C. After washing 100 μl of substrate CellTiter-Glo (Promega, Madison, WI, USA) were added per well, and the bioluminescence measured using a Tecan Infinite F200 plate-reading luminometer (Tecan Trading AG, Switzerland). Experiments were performed in triplicates.

### Cell viability assay

For growth inhibition assays, A431 cells were seeded at a density of 2,000 cells/well in 96-well plates in DCM. After 24 hours, medium was then replaced by DMEM supplemented with 1% v/v FCS containing purified antibodies: cetuximab (0.07–4.5 μg/ml), muromonab (0.07–4.5 μg/ml), ttαCEA (0.1–3 μg/ml) or ttαGFP-αEGFR-αCEA (0.1–3 μg/ml). Cell were incubated for 72 hours in humidified 5% CO_2_ atmosphere at 37 °C, after which cell viability was assessed using the CellTiter-Glo luminescent assay and the bioluminescence measured using a Tecan Infinite F200 plate-reading luminometer. Experiments were performed in triplicates.

### Circular Dichroism (CD)

Circular dichroism measurements were performed with a Jasco J-810 spectropolarimeter (JASCO, Tokyo, Japan). The spectra were recorded on protein samples at 0.03 or 0.05 g/L in PBS using 0.2 cm path length quartz cuvettes at 25 °C. Thermal denaturation curves from 10 to 95 °C were recorded on the same protein samples and cuvette by increasing temperature at a rate of 1 °C/minute and measuring the change in ellipticity at 210 nm. The reported mid-point temperature of protein denaturation corresponds to the minima (or maxima) in the corresponding derivative curve.

### Size exclusion chromatography-multiangle laser light scattering (SEC-MALLS)

Static light scattering experiments were performed at room temperature using a Superdex 200 10/300 GL column (GE HealthCare) attached in-line to a DAWN-HELEOS light scattering detector and an Optilab rEX differential refractive index detector (Wyatt Technology, Santa Barbara, CA, USA). The column was equilibrated with running buffer (PBS + 0.03% NaN_3_, 0.1 μm filtered) and the SEC-MALLS system was calibrated with a sample of BSA at 1 g/L in the same buffer. Then 100 μL samples of the different antibodies at 0.1–0.8 g/L in PBS were injected into the column at a flow rate of 0.5 mL/min. Data acquisition and analysis were performed using ASTRA software (Wyatt Technology). The molar mass data shown in the figures were smoothed (the mass measured at the center of the peak changes by less than 0.5% after smoothing). Based on numerous measurements on BSA samples at 1 g/L under the same or similar conditions we estimate that the experimental error in the molar mass is around 5%.

### Mass spectrometry

A 2 μl protein sample was desalted using ZipTip® C4 micro-columns (Millipore) and eluted with 0.5 μl SA (sinapinic acid, 10 mg/ml in [70:30] Acetonitrile: Trifluoroacetic acid 0.1%) matrix onto a GroundSteel massive 384 target (Bruker Daltonics, Billerica, MA, USA). An Autoflex III MALDI-TOF/TOF spectrometer (Bruker Daltonics) was used in linear mode with the following settings: 5000–40000 Th window, linear positive mode, ion source 1: 20 kV, ion source 2: 18.5 kV, lens: 9 kV, pulsed ion extraction of 120 ns, high gating ion suppression up to 1000 Mr. Mass calibration was performed externally with protein 1 standard calibration mixture (Bruker Daltonics). Data acquisition, peak peaking and subsequent spectra analysis was performed using FlexControl 3.0 and FlexAnalysis 3.0 software (Bruker Daltonics).

### Kinetic measurements using biolayer interferometry

The antigen binding kinetics of αCEA^N7^ and ttαCEA trimerbodies was performed by loading three AR2G biosensors (Fortebio, Menlo Park, CA, USA) with either purified trimerbody at 10 μg/ml in loading buffer (10 mM acetate, pH 6), for 30 minutes, to a signal of 4.69 ± 0.06 nm for αCEA^N7^ and 2.95 ± 0.03 nm for ttαCEA. Following re-equilibration in kinetics buffer (0.1% BSA in PBS, 0.05% Tween20), association was measured over 2 hours against 50, 25 and 12.5 nM of CEA diluted in kinetics buffer, followed by 2 hours of dissociation in analyte-free kinetics buffer. The experiments were performed at 30 °C on an Octet RED96 instrument (Fortebio) while shaken at 1000 rpm. Sensorgrams obtained from individual biosensors were analyzed by local fitting to a 1:1 model with Octet Data Analysis software (Fortebio). The multispecificity of ttαGFP-αCEA^(1:2)^ and ttαGFP-αEGFR-αCEA trimerbodies were demonstrated by measuring their simultaneous binding of multiple antigens. First, GFP was immobilized onto AR2G biosensors at 10 μg/mL in loading buffer, for 20 minutes, to a signal of 0.77 ± 0.12 nm. The GFP-coated biosensors were loaded with ttαGFP-αCEA^(1:2)^ or ttαGFP-αEGFR-αCEA trimerbodies at 30 nM for 1 hour in kinetics buffer. The trimerbody-loaded biosensors were then moved into wells containing 50 nM of CEA in kinetics buffer for 1 hour. Finally, the biosensors were moved into wells with 10 nM of EGFR in kinetics buffer for 1 hour, followed by 1 hour of dissociation in analyte-free kinetics buffer. For every step involving analyte (e.g. trimerbody, CEA, and EGFR), a control biosensor was included which was moved into an analyte-free well, and a trimerbody-free biosensor was used to monitor the non-specific binding of CEA and EGFR to the GFP-coated biosensor surface. BSA-coated biosensors gave no response against 30 nM of ttαGFP-αCEA^(1:2)^ or ttαGFP-αEGFR-αCEA trimerbodies in kinetics buffer (data not shown).

### Analysis of the active surface

The comparative analysis of the sampling space accessible for the V_HH_ domains in the intermolecular versus the intramolecular (single chain) trimerbodies was performed through homology modeling with MODELLER^36^. The crystal structure of human collagen XVIII trimerization domain^16^ deposited in the Protein Data Bank^37^ with entry 3HSH was used as template for the trimerization domain, and the crystal structure of one of the Fab fragments in PDB entry 2VXS^38^ for the V_HH,_ as previously described^10^. A total of 150 highly optimized models where build for each trimerbody, in which the relative position between the V_HH_ and the trimerization domains was left loose, thus allowing the exploration of multiple configurations for the flexible linkers. All the models where structurally aligned through the trimerization domain and the surface of all of them was merged and evaluated with Pymol^39^.

### Statistical Analysis

Experiments were done in triplicates and results were expressed as mean ± standard deviation (SD). The data were evaluated usingthe Student’s t-test. Statistical analysis was performed using Prism (GraphPad Software, San Diego, CA).

## Additional Information

**How to cite this article**: Alvarez-Cienfuegos, A. *et al*. Intramolecular trimerization, a novel strategy for making multispecific antibodies with controlled orientation of the antigen binding domains. *Sci. Rep*. **6**, 28643; doi: 10.1038/srep28643 (2016).

## Supplementary Material

Supplementary Information
