# Supplementary material for: Intramolecular trimerization, a novel strategy for making multispecific antibodies with controlled orientation of the antigen binding domains
Source: Sci Rep. 2016 Jun 27;6:28643. doi: 10.1038/srep28643 (PMC4921811; doi:10.1038/srep28643)
Supplement: Supplementary Information [file srep28643-s1.pdf]

<u>**Supplementary information**</u>

Intramolecular trimerization, a novel strategy for making multispecific antibodies with controlled orientation of the antigen binding domains

Ana Alvarez-Cienfuegos, Natalia Nuñez-Prado, Marta Compte, Angel M. Cuesta, Ana Blanco-Toribio, Seandean Lykke Harwood, Maider Villate, Nekane Merino, Jaume Bonet, Rocio Navarro, Clara Muñoz-Briones, Karen Marie Juul Sørensen, Kasper Mølgaard, Baldo Oliva, Laura Sanz, Francisco J. Blanco, Luis Alvarez-Vallina

Supplementary Table S1. Kinetic constants from biolayer interferometry

**Molecule**	$\mathbf{K_D}$ (nM)	$\mathbf{k_a}$ $(M^{-1}s^{-1})$	$\mathbf{k_d}$ (s^{-1})
**αCEA^{N7}**	12.0	$1.23 * 10^{4}$	$1.48 * 10^{-4}$
**ttαCEA**	11.8	$1.14 * 10^{4}$	$1.34 * 10^{-4}$

Kinetic constants for αCEA^{N7} and ttαCEA were obtained from global fitting of sensorgrams. The presented values are derived from 3 biosensors for each molecule.

Supplementary Figure 1. Full chromatograms corresponding to figure 6 and SDS-PAGE analysis

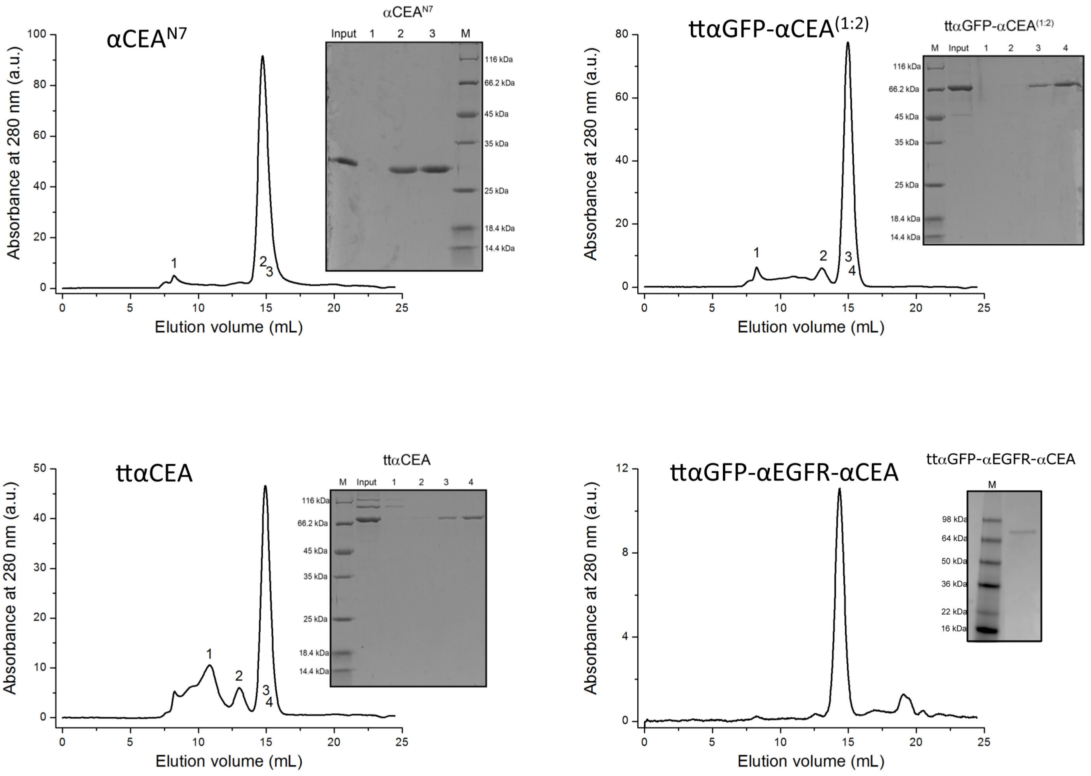

The injected sample (Input), some of the collected fractions (lanes 1 to 4) and molecular weight markers (M) were run on a 12 % acrylamide gel and stained with coomassie blue. The numbers on the chromatogram correspond to the position of the fractions that were loaded on the numbered SDS-PAGE lanes. For ttαGFP-αEGFR- αCEA the fractions contained too little protein to be detected in the gel under the same conditions as used for the other proteins and only the input is shown. The Exclusion volume of the column (estimated by the elution volume of bromophenol blue) is 8.6 mL. The chromatograms show a major peak at 14.4-15.0 mL corresponding to the timerbody. There is also a very small peak at around 8.3 mL in all four chromatograms that might contain very large aggregates. In the case of ttαCEA trimerbody there are additional minor peaks at 11 mL and 13 mL. At least the first one corresponds to high molecular weight contaminant proteins, as seen in lane 1 of the corresponding SDS-PAGE.

Supplementary Figure 2. Circular dichroism spectra of purified multi-chain and tandem trimerbodies

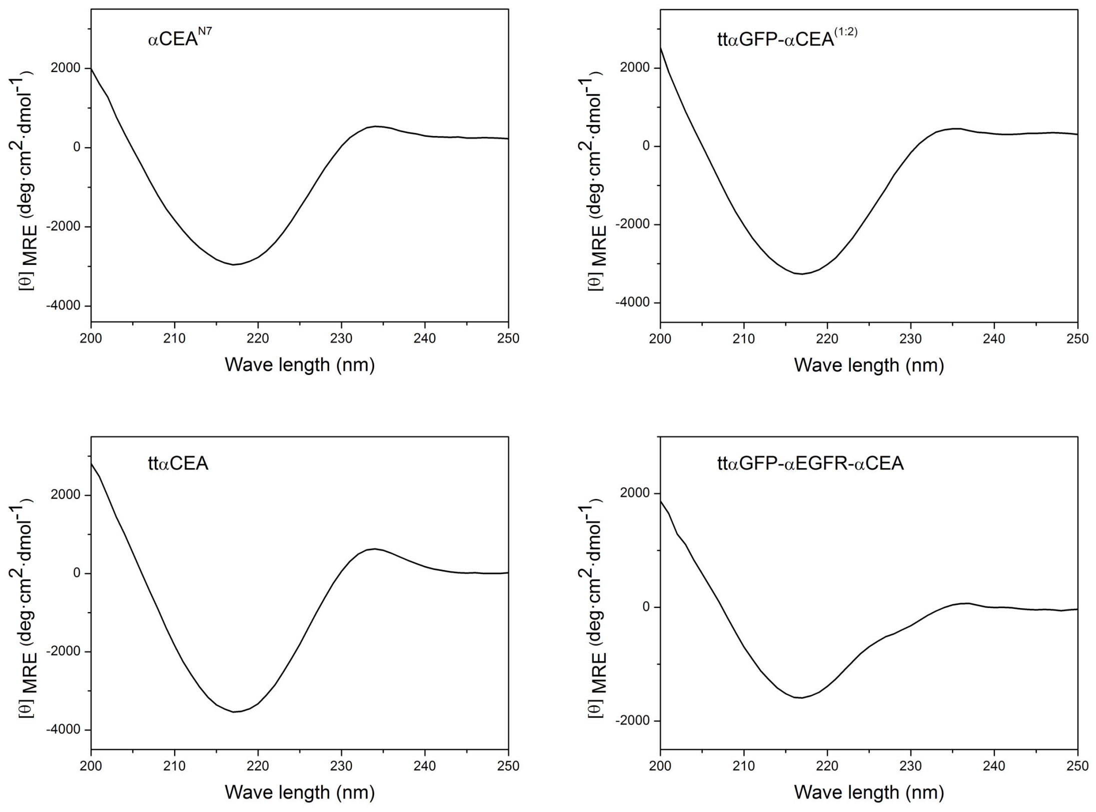

All spectra were recorded at 25 °C on samples 0.05 g/L, except for ttαGFP-αEGFR-αCEA (0.03 g/L).

Supplementary Figure 3. Serum stability of purified αCEA^{N7}, ttαCEA and ttαGFP-αCEA$^{(1:2)}$ trimerbodies

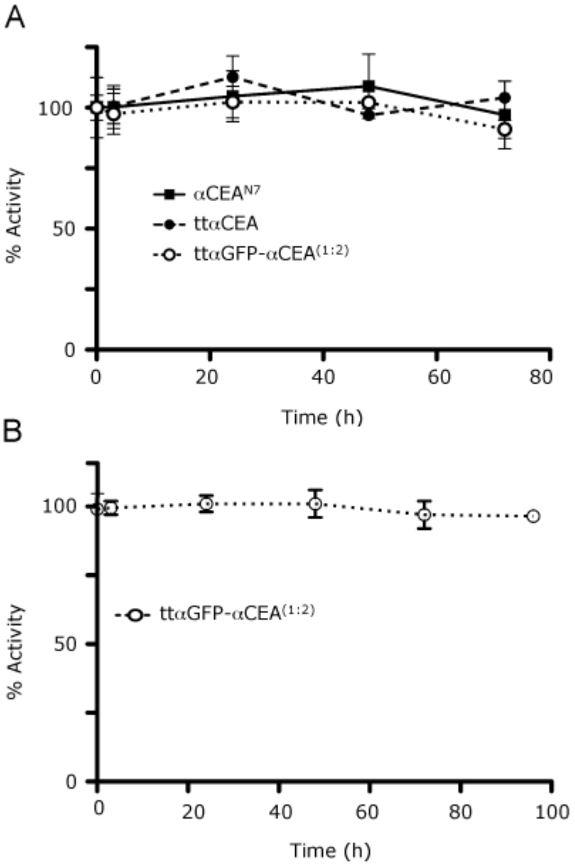

ELISA against plastic immobilized human CEA (A) or GFP (B) was performed after incubation at 37 °C for different time periods in human serum, as explained in material and methods.
